# Human β-defensin 3 contains an oncolytic motif that binds PI(4,5)P_2_ to mediate tumour cell permeabilisation

**DOI:** 10.18632/oncotarget.6520

**Published:** 2015-12-09

**Authors:** Thanh Kha Phan, Fung T. Lay, Ivan K.H. Poon, Mark G. Hinds, Marc Kvansakul, Mark D. Hulett

**Affiliations:** ^1^ Department of Biochemistry and Genetics, La Trobe Institute for Molecular Science, La Trobe University, Melbourne, Victoria, 3086, Australia; ^2^ Department of Chemistry and Physics, La Trobe Institute for Molecular Science, La Trobe University, Melbourne, Victoria, 3086, Australia

**Keywords:** HBD-3, defensin, necrosis, tumour cell lysis, PI(4,5)P_2_

## Abstract

Cationic antimicrobial peptides (CAPs), including taxonomically diverse defensins, are innate defense molecules that display potent antimicrobial and immunomodulatory activities. Specific CAPs have also been shown to possess anticancer activities; however, their mechanisms of action are not well defined. Recently, the plant defensin NaD1 was shown to induce tumour cell lysis by directly binding to the plasma membrane phosphoinositide, phosphatidylinositol 4,5-bisphosphate (PI(4,5)P_2_). The NaD1–lipid interaction was structurally defined by X-ray crystallography, with the defensin forming a dimer that binds PI(4,5)P_2_ via its cationic β2-β3 loops in a ‘cationic grip’ conformation. In this study, we show that human β-defensin 3 (HBD-3) contains a homologous β2-β3 loop that binds phosphoinositides. The binding of HBD-3 to PI(4,5)P_2_ was shown to be critical for mediating cytolysis of tumour cells, suggesting a conserved mechanism of action for defensins across diverse species. These data not only identify an evolutionary conservation of CAP structure and function for lipid binding, but also suggest that PIP-binding CAPs could be exploited for novel multifunction therapeutics.

## INTRODUCTION

Naturally occurring antimicrobial peptides (AMPs) are an important component of host innate immunity, representing the first line of chemical defense against invading pathogens. Defensins are a prominent family of cationic AMPs (CAPs) that are ubiquitously expressed in plant, fungi, invertebrates and vertebrates. Despite limited amino acid sequence identities and different disulfide frameworks (6–10 cysteines) amongst defensin subfamilies, their tertiary structures share remarkably similar folds that feature a triple-stranded antiparallel β-sheet (or equivalent pattern thereof) constrained by intramolecular disulfide bridges [1–3]. Defensins and many other CAPs display diverse functions and mechanisms of action, including multimodal and multi-target microbicidal effects, and immunomodulatory activities [3–7]. Additionally, a number of CAPs have been found to exhibit specific anticancer activity against solid and/or hematological tumours, and hence represent a potential therapeutic strategy to counter current issues of adverse side-effects and multidrug resistance [8–10].

*Nicotiana alata* defensin 1 (NaD1), a potent antifungal peptide from the flowers of the ornamental tobacco, was reported to selectively kill a broad spectrum of tumour cells *in vitro* at low micromolar concentrations [11]. The underlying mechanism was described to involve the entry of NaD1 into the cell followed by binding to PI(4,5)P_2_, leading to membrane permeabilisation, membrane blebbing and eventually to cell lysis [11]. Similarly, Baxter *et al.* [12] recently demonstrated PI(4,5)P_2_ specificity and tumour cell cytotoxicity for the related tomato defensin TPP3, suggesting a shared molecular target and mechanism of action for these defensins.

PI(4,5)P_2_ is one of seven phosphorylated derivatives of phosphatidylinositol, which are collectively known as PIPs. Despite their low abundance, they play important regulatory roles for diverse cellular processes, including cellular signaling, cytoskeletal rearrangement and membrane trafficking [13–15]. NaD1 and TPP3 have been shown to bind PI(4,5)P_2_ via their cysteine-flanked highly-positively charged β2-β3 loop (residues 36–40 in NaD1 and residues 38–42 in TPP3) [11, 12]. As a dimer, two β2-β3 loops of NaD1 monomers form a claw-like structure with PI(4,5)P_2_ accomodated in the binding grip. The protein-lipid interaction involves an intensive H-bonding network provided by residues within and around the β2-β3 loop. Defects in PI(4,5)P_2_ binding effectively lead to severe impairment of the anticancer activity of NaD1 [11]. Equivalently, the importance of the β2-β3 loop, in PI(4,5)P_2_ binding was also recently reported for TPP3 [12].

The β2-β3 loop of NaD1 and TPP3 is highly conserved among class II defensins of solanaceous plants, and interestingly, is also shared with human β-defensin 3 (HBD-3). HBD-3 is inducibly expressed and secreted by epithelial cells, several non-epithelial tissues, monocytes and neutrophils [16–19] and is arguably the most potent antimicrobial of the β-defensins [20–22]. HBD-3 exhibits broad-spectrum antibacterial, antifungal and antiviral activities [16, 17, 23–27]. HBD-3 is also chemoattractive and activates antigen presenting cells as well as induces chemokine expression, crucially contributing to the integration of innate and adaptive immune responses [28–31]. HBD-3 has been proposed to interact with bacterial lipid II [32], monocytic phosphatidylserine [33], and different subsets of Toll-like, CC and CXC chemokine receptors [30, 31, 34, 35]. However, it should be noted that the biological involvement of many of these targets in HBD-3 activities has been challenged in recent years [36]. In addition, an anti-metastatic effect on head, neck and colon tumour cells has been reported [37, 38], although the anticancer mechanism of HBD-3 remains poorly defined.

In this report, we demonstrate for the first time that a human CAP, HBD-3, binds phosphoinositides and that the interaction with PI(4,5)P_2_ in particular, is critical for the tumour cell killing activity of this defensin. Our data support the importance of a cationic β2-β3 loop for PIP binding, that contributes to a conserved mechanism of tumour cell/pathogen cytolysis among innate molecules with NaD1-like ‘cationic-grip’ dimeric structures. This study identifies PIP-binding CAPs as a potential new generation of multifaceted therapeutics, particularly as anticancer agents.

## RESULTS

### HBD-3 shares a conserved β2-β3 loop motif with the plant defensins NaD1 and TPP3

**Figure 1:**
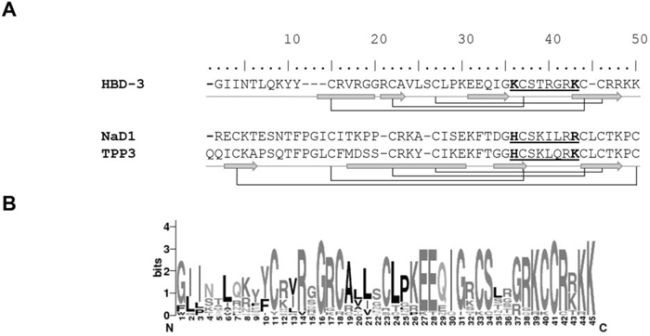
Structural conservation of HBD-3, NaD1 and TPP3 **A.** Schematic illustration of mature defensin secondary structures. HBD-3, NaD1 and TPP3 were aligned according to conserved cysteine residues, which form intramolecular disulfide bonds (connecting lines). Secondary structures (α-helix, block; β-strand, arrow), cationic β2-β3 (underlined) and conserved residues of interest (bold) are shown explicitly. **B.** Sequence logo of mammalian β-defensin 3, based on sequence alignment of HBD-3 and homologues using WebLogo (http://weblogo.berkeley.edu).

Although HBD-3, NaD1 and TPP3 share relatively low sequence identity and differ in disulfide connectivity patterns and secondary structure arrangement, conservation of their cysteine-flanked cationic β2–β3 loops (ST**R**G**RK**, S**K**IL**RR** and S**K**LQ**RK** respectively), including overall loop charge (+3), and basic residue arrangement are apparent (Figure 1A). Also, K32 that precedes the loop in HBD-3 is the equivalent to H33 in NaD1 and H34 in TPP3 as a potential H-bond donor. Furthermore, the homology of the β2-β3 loop as well as the occurrence of potential H-bonding residues (R, K, S or T) at position 32 is also observed amongst other mamalian homologues (Figure 1B), implying a functional importance.

### HBD-3 is selectively cytolytic to tumour cells via bleb-associated membrane permeabilisation

**Figure 2:**
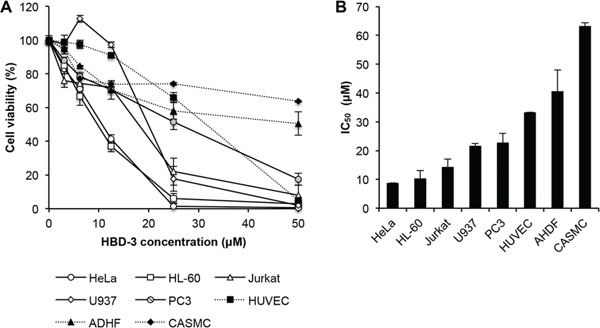
Tumour cell-selective cytotoxicity of HBD-3 **A.** Dose-dependent cytotoxicity and **B.** estimated IC_50_ value of HBD-3, on different tumour (HeLa, HL-60, Jurkat, U937 and PC3) and normal primary (HUVEC, ADHF and CASMC) cell lines, determined by tetrazolium-coupled cell viability assay. Data were normalised against untreated control, which was arbitrarily assigned as 100% cell viability. Data represent mean ± SEM of three independent experiments.

To investigate the cytotoxic effects of HBD-3 on cell viability, tetrazolium-based assays were performed on a number of different human tumour and primary cell lines. HBD-3 showed dose-dependent cytotoxicity on tumour lines HeLa, HL-60, Jukat, U937 and PC3, at low micromolar concentrations with IC_50_ values from 8–20 μM (Figure 2). Primary cells, HUVEC and particularly AHDF and CASMC, were less susceptible to HBD-3 treatment (IC_50_ values from 30–65 μM). No discrimination between adherent and suspension cells was observed.

**Figure 3:**
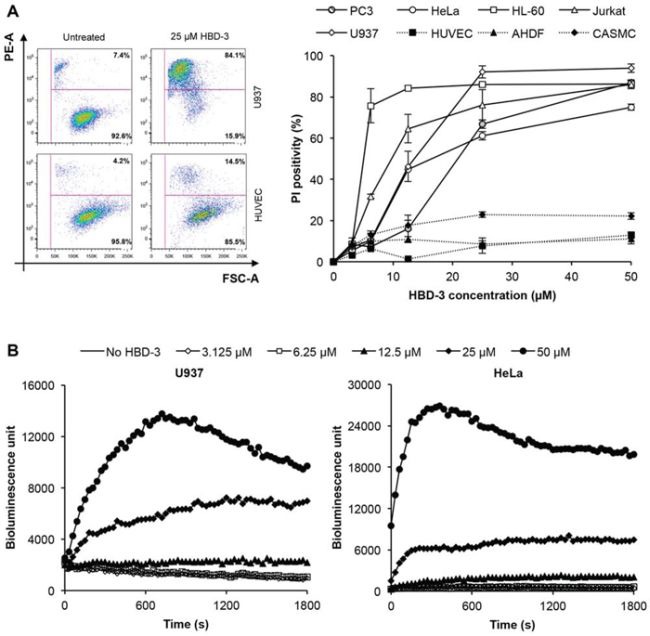
Tumour cell-selective membrane permeabilisation by HBD-3 **A.** Flow cytometry-based PI uptake assay of different tumour and primary cell lines treated with or without HBD-3. Cells were gated based on forward scatter (FSC-A) and side scatter (SSC-A). Level of permeabilisation was expressed as PI-positivity, detected by PE-A signal. Data represent mean ± SEM of three independent experiments. **B.** ATP bioluminescence assay of U937 and HeLa cells treated with HBD-3 titrations. Level of cytoplasmic ATP released was detected as bioluminescence emission signal intensity. Data are representative of three independent experiments.

Flow cytometry-based assays using the membrane impermeable dye PI were then conducted to examine the ability of HBD-3 to induce membrane permeabilisation. HBD-3 was able to permeabilise all cell types tested to varying levels in a dose-dependent manner (Figure 3A). Upon HBD-3 treatment at concentrations higher than 10 μM, the tumour cells were more readily permeabilised than the primary cells. For example, approximately 85% of U937 cells, compared to 14.5% of HUVEC cells, treated with 25 μM HBD-3 were PI-positive. Even at the highest dose tested (50 μM HBD-3), less than 20% of cells for each primary cell type compared to at least 80% of cells for the tumour lines, were permeabilised. Furthermore, cytoplasmic ATP was rapidly released from U937 and HeLa cells by HBD-3 in a similar concentration-dependent manner (Figure 3B). Together, these data suggest that HBD-3 causes tumour cell death via rapid, and somewhat selective, membrane permeabilisation.

**Figure 4:**
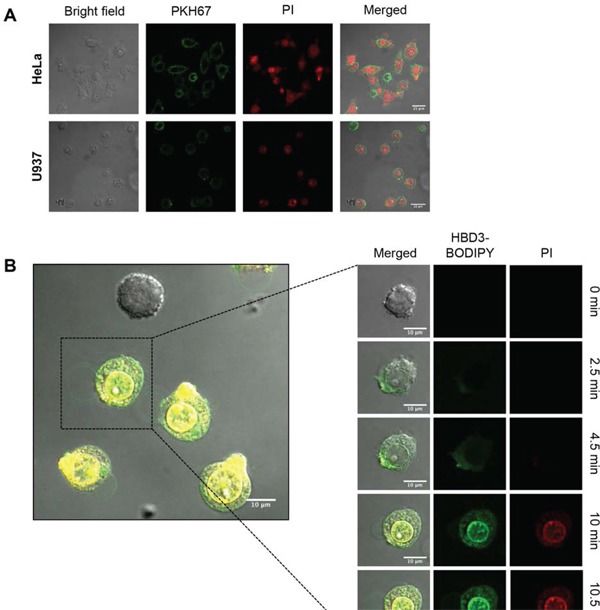
Bleb-associated membrane permeabilisation of HBD-3 **A.** CLSM imaging of PKH67-prestained HeLa and U937 cells at 15 min after addition of 15 μM HBD-3. **B.** Time-lapse CLSM imaging of U937 cells treated with 15 μM HBD-3-BODIPY FL EDA. Scale bars represent 10 μm. Data in A and B are representative of three independent experiments.

Using confocal laser scanning microscopy (CLSM), the morphology of HBD-3 treated U937 and HeLa cells was visualised in the presence of the membrane stain PKH67 and permeabilisation indicator PI. HBD-3 induced the formation of large membrane blebs (>10 μm) in the PI-positive cells (Figure 4A). To further monitor the kinetics and localisation of membrane permeabilisation process, a time-course CLSM study using BODIPY FL-labeled HBD-3 was performed on U937 cells (Figure 4B). Initially, at 2.5 min post-addition, HBD-3 accumulated locally on the outer plasma membrane, before gaining entry through the accumulation point. HBD-3 then appears to target and bind to the inner membrane leaflet, and potentially other cytoplasmic membranes, including the nuclear envelope at 4.5 min. PI staining, indicating membrane disruption, subsequently coincided with initiation of membrane blebbing. PI signal intensification and bleb expansion were further observed from 10 min onward. No membrane accumulation of HBD-3 and bleb formation was detected on viable (PI-negative) cells.

### HBD-3 binds to PI(4,5)P_2_ *in vitro* and in cellular membranes of tumour cells

**Figure 5:**
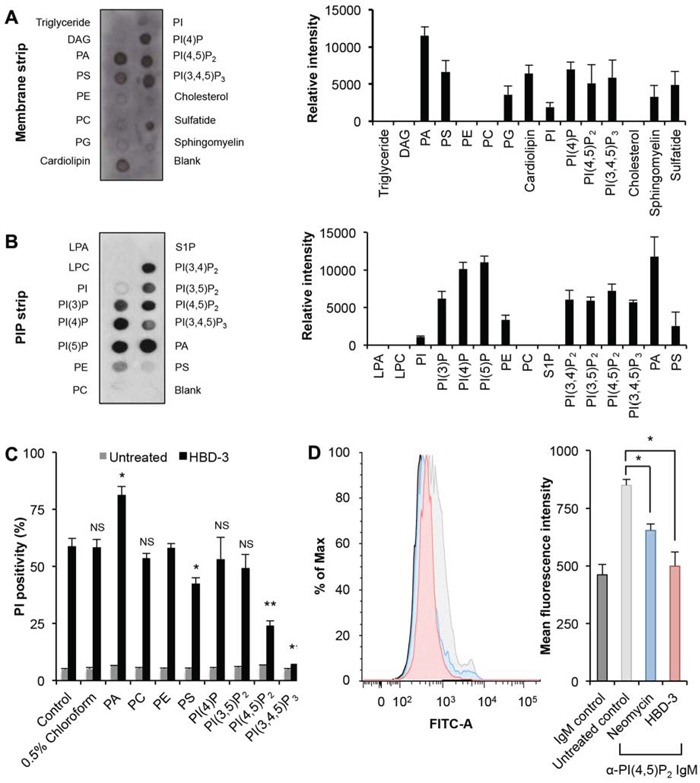
Binding to phospholipids by HBD-3 Immunodetection of lipid binding by HBD-3 on a **A.** membrane strip and **B.** PIP strip. Relative binding intensity was determined by densitometry analysis of chemiluminescence signals. **C.** Inhibitory effect of lipids on HBD-3 mediated membrane permeabilisation. HBD-3 was pre-incubated with lipids prior to flow cytometry-based PI uptake assay. **D.** Blocking of anti-PI(4,5)P_2_ antibody binding to plasma membrane PI(4,5)P_2_ by HBD-3. Untreated and treated U937 cells were fixed, lysed and stained with FITC-conjugated anti-PI(4,5)P_2_ IgM prior to flow cytometry analysis. Data in A–D represent mean ± SEM of three independent experiments. NS, no significant; *, *p* < 0.05; **, *p* < 0.01; unpaired *t*-test.

The anticancer activity of NaD1 was shown to mediated by its interaction with PIPs, particularly PI(4,5)P2 [11]. Therefore, protein-lipid overlay assays using commercially available MembraneTM (Figure 5A) and PIPTM (Figure 5B) strips were performed to study the binding of HBD-3 to different functionally important lipids. Among membrane lipids, HBD-3 showed greater relative binding intensity toward phospholipids, especially PA, PIPs including (PI(4)P, PI(4,5)P2 and PI(3,4,5)P3), PS, cardiolipin, and, to a lesser extent, PG. Weaker interactions with sphingolipids (sphingomyelins and sulfatide) were also detected. The PIP™ strip further indicated the binding preference of HBD-3 towards, most strongly, PA and all phosphoinositides, but not other lipids, including phosphatidylinositol.

To confirm the relevance of these HBD-3:lipid interactions on the ability of HBD-3 to permeabilise tumour cell membranes, lipid inhibition of HBD-3 mediated PI uptake by U937 cells was examined. Among all tested lipids, PI(4,5)P_2_ and PI(3,4,5)P_3_ showed the greatest inhibitory activity, with ∼50% and 100% reduced PI-positivity, respectively (Figure 5C). A much lower inhibition was also observed for PS, with ∼15% reduction. Other lipids, including PC, PE, and particularly PI(4)P and PI(3,5)P_2_, did not show any significant inhibition. Interestingly, PA, the lipid suggested to bind most strongly to HBD-3 by lipid protein overlay assays, did not show any inhibitory activity but instead enhanced permeabilisation.

A flow cytometry-coupled immunodetection assay using FITC-conjugated anti-PI(4,5)P_2_ IgM was then used to determine if HBD-3 interacted with PI(4,5)P_2_ on tumour cell membranes. This assay was based on the premise that the interaction of HBD-3 with PI(4,5)P_2_ would block antibody binding to the lipid at inner plasma membrane leaflet. Indeed, HBD-3 blocked anti-PI(4,5)P_2_ antibody binding to fixed and membrane permeabilised cells, as indicated by the significant decrease of overall fluorescence intensity, compared to the untreated control (Figure 5D). Neomycin, a well-documented PI(4,5)P2 sequestering molecule [39–42], showed similar blocking of anti-PI(4,5)P2 antibody binding as HBD-3. An isotype control was also included to indicate background binding (Figure 5D).

### Interaction with PI(4,5)P_2_ is required for HBD3-induced membrane permeabilisation

**Figure 6:**
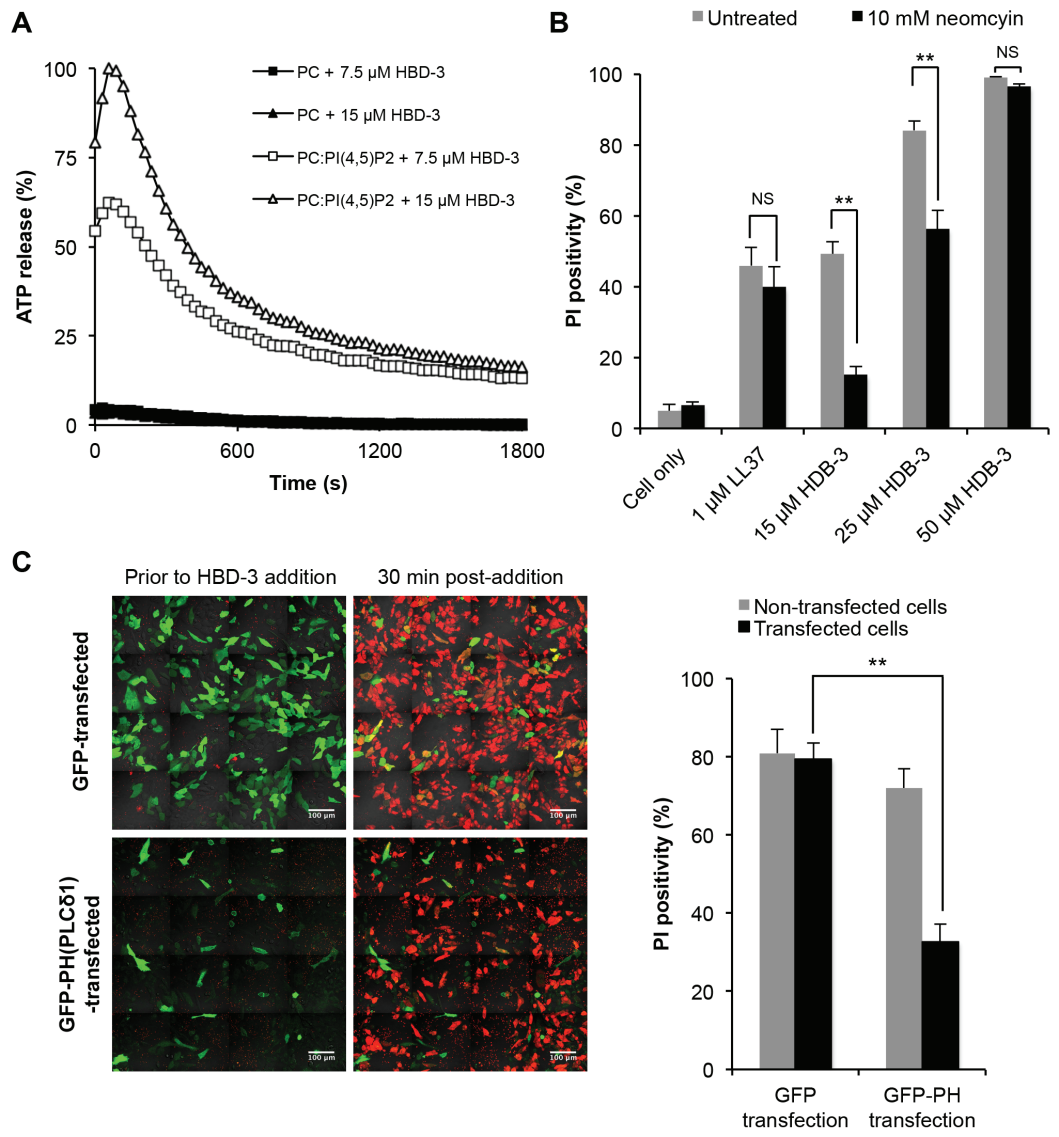
Importance of PI(4,5)P2 binding in HBD3-mediated membrane permeabilisation **A.** HBD-3 induced ATP release of ATP-encapsulated PC and PC:PI(4,5)P_2_ liposomes. Level of ATP released was detected as bioluminescence signals and normalised against HEPES only (background) and Triton-X 100 treated (100% lysis) control. Data are representative of three independent experiments. **B.** Inhibitory effect of neomycin on membrane permeabilisation by HBD-3. U937 cells were treated with neomycin prior to flow cytometry-based PI uptake assay. Data represent mean ± SEM of three independent experiments. **C.** Inhibitory effect of PH domain on on membrane permeabilisation by HBD-3. GFP and GFP-PH transfected HeLa cells were treated with HBD-3, followed by confocal imaging and PI-positive counting. Data represent mean ± SEM of five independent experiments, each with three field of view containing at least 30 (GFP-PH) or 100 transfected cells (GFP only). NS, no significant; *, *p* < 0.05; **, *p* < 0.01; unpaired *t*-test.

To investigate the potential relationship between PI(4,5)P_2_ binding and HBD-3-mediated membrane permeabilisation, liposome leakage assays were conducted by measuring the release of encapsulated ATP by bioluminescence. Whilst HBD-3 minimally induced ATP release from ATP-encapsulated PC only liposomes, a much higher level of ATP was released from PC liposomes containing 5% PI(4,5)P_2_. This liposome permeabilisation effect was concentration-dependent, as increasing ATP release levels were observed with increasing HBD-3 concentration (Figure 6A).

The importance of PI(4,5)P_2_ binding was further demonstrated by the competitive inhibition of HBD-3 mediated membrane permeabilisation with neomycin (Figure 6B). Compared to the untreated control, U937 cells pre-incubated with neomycin were significantly less susceptible to HBD-3 treatment, but not with LL-37, another CAP that exhibits non-PI(4,5)P_2_-dependent detergent-like [43–45] and/or pore-forming [46] membrane disruption. However, the inhibitory effect of neomycin was overcome at higher concentrations of HBD-3, suggesting competition between neomycin and HBD-3 for PI(4,5)P_2_ binding. Similarly, blocking PI(4.5)P_2_ by endogenously overexpressing a GFP-tagged PI(4,5)P_2_-sequestering PH domain from PLC(δ) also caused reduced HBD-3 activity as demonstrated by the 2.5-fold lower number of PI-positive HeLa cells upon HBD-3 treatment compared to GFP only-transfected cells (Figure 6C).

### Mutation of conserved residues in HBD-3 leads to decreased PI(4,5)P_2_ binding

**Figure 7:**
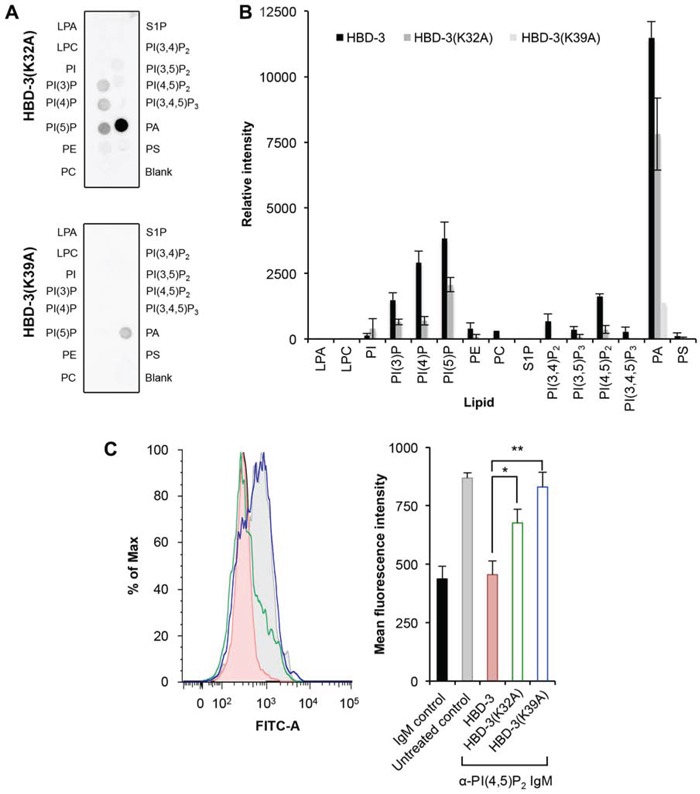
Reduced lipid binding by HBD-3 mutants **A.** Immunodetection of lipid binding by HBD-3 mutants to lipids on a PIP strip. **B.** Densitometry analysis of (A) in comparison with HBD-3 (strips processed simultaneously, not shown). **C.** Blocking of anti-PI(4,5)P_2_ antibody binding to plasma membrane PI(4,5)P_2_ by HBD-3 and mutants. Data in (B) and (C) represent mean ± SEM of three independent experiments. *, *p* < 0.05; **, *p* < 0.01; unpaired *t*-test.

The anticancer activity and PI(4,5)P_2_ binding of NaD1 and TPP3 was mapped to a cationic loop region between strands β2 and β3 [11, 12]. As described, HBD-3 also possesses a β2-β3 loop with a similar stretch of basic residues to NaD1 and TPP3. Most importantly, residues K32 and K39 on HBD-3 are respectively equivalent to H33 and R40 in NaD1, and H34 and K41 in TPP3. These residues act as H-bond donors in NaD1 and, potentially, in TPP3 with the phosphate groups of PI(4,5)P_2_. Furthermore, R40 of NaD1 and K41 of TPP3 have been propsed as critical for cooperative PI(4,5)P_2_ binding (i.e. linking multiple PI(4,5)P_2_ molecules) and establishing plant defensin-PI(4,5)P_2_ oligomerisation. To study the importance of these residues in HBD-3, alanine mutants were generated and subjected to lipid binding and membrane permeabilisation assays. Using PIP™ strips (Figure 7A and 7B), HBD-3(K32A) was showed to maintain the lipid binding specificity of wild-type HDB-3 but at much lower levels; the exception being its interaction with PA that was substantially retained. In contrast, HBD-3(K39A) showed a pronounced loss of PIP binding while maintaining binding to PA. The importance of K32 and K39 to the binding of PI(4,5)P_2_ was also specifically demonstrated by the reduced ability of alanine mutants to block anti-PI(4,5)P_2_ antibody binding to U937 cells when compared to wild-type HBD3-treated cells. This was more evident for HBD-3(K39A) as anti-PI(4,5)P_2_ antibody binding to treated cells was essentially the same as that of the untreated control (Figure 7C).

### HBD-3 mutants show impaired tumour cell cytotoxicity and cytolysis

**Figure 8:**
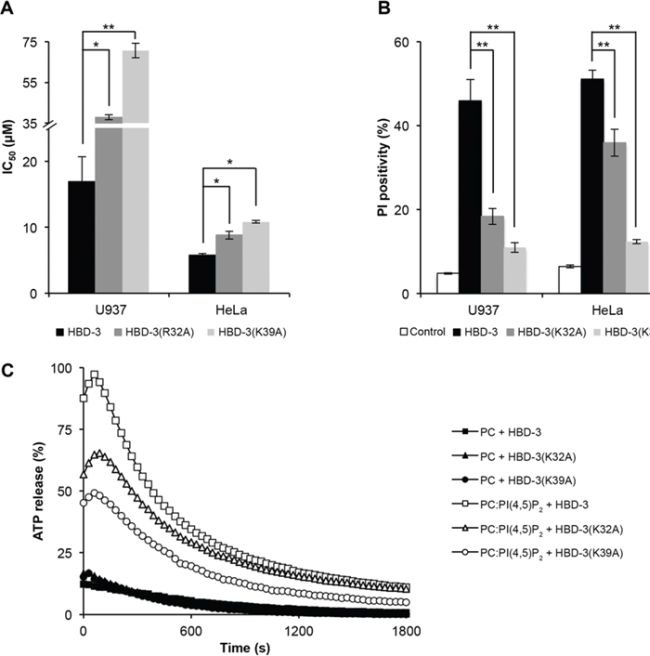
Impaired tumour cell killing and membrane permeabilisation by HBD-3 mutants **A.** IC_50_ values (by tetrazolium-coupled cell viability assay) and **B.** membrane permeabilisation effect (by flow cytometry-based PI uptake assay) of HBD-3 and its mutants on U937 and HeLa cells. Data in (B) and **C.** represent mean ± SEM of three independent experiments. *, *p* < 0.05; **, *p* < 0.01; unpaired *t*-test. (C) ATP release of ATP-encapsulated PC ± PC:PI(4,5)P_2_ liposomes by 15 μM HBD-3 and its mutants. Data are representative of three independent experiments.

Cell viability assays revealed that HBD-3(K32A) and HBD-3(K39A) had reduced tumour cell cytotoxicity, compared to wild-type HBD-3. HBD-3(K39A) displayed the greatest impairment, with three-fold and two-fold higher IC_50_ for U937 and HeLa cells, respectively (Figure 8A). Similarly, there was a reduction in the ability of the HBD-3 mutants to permeabilise U937 or HeLa cells as measured by PI uptake. The reduced ability of each mutant to permeabilise cells was consistent with the cell viability results (Figure 8B). Furthermore, the ability HBD-3(K32A) or HBD-3(K39A) to induce ATP release from ATP-encapsulated PC:PI(4,5)P_2_ liposomes was also impaired in similar manner (Figure 8C).

## DISCUSSION

CAPs, of which defensins are a major family, are important innate defense and immunomodulatory molecules, employed by most living organisms to combat pathogenic invasions and orchestrate host immune responses. In addition, a number of defensins and other CAPs have also recently been suggested to have anticancer activity. HBD-3 of the human β-defensin subfamily displays potent activity against fungi, bacteria and viruses, as well as acts as an indespensable effector of immunity [16, 17, 23–31]. However, the precise molecular basis underlying these functions of HBD-3, particularly the anticancer activity, remains unclear. Indeed, to date, HBD-3 has only been shown to inhibit tumour cell migration by suppressing vascular endothelial growth factor in head and neck cancer cells [37] or down-regulating MTA2 (metastasis-associated 1 family, member 2) expression of colon cancer cells [38].

In this study, we report the selective tumour cell-killing activity of HBD-3 against a broad spectrum of epithelial and haemotological tumour cell lines at low micromolar concentrations, via bleb-associated membrane permeabilisation and cell lysis. We have identified PIPs as lipid targets, such as PI(4,5)P_2_ on the plasma membrane, to which HBD-3 binds and exerts its membrane disrupting effect. It is likely that HBD-3 tumour cell cytotoxicity is initiated by local concentration of HBD-3 at the cell surface, possibly by electrostatic attraction, leading to plasma membrane weakening at accumulation point(s), which allows HBD-3 internalisation. Once it enters the cytoplasm, HBD-3 binds to PI(4,5)P_2_ on the inner leaflet of the plasma membrane, and possibly to other PIPs of subcellular organelle membranes, causing membrane disruption, bleb formation and ultimately cell lysis.

Many CAPs have previously been shown to selectively kill tumour cells by various mechanisms. For example, melittin, an α-helical CAP from European honeybee (*Apis mellifera*), is lytic to human leukaemic tumour cells, primarily by inserting into the membrane bilayer and forming barrel-stave pores [47–49]. Bovine lactoferricin triggers either necrotic membrane disruption or apoptosis depending on the target cell type, and also inhibits xenografted tumour growth and metatasis [50–54]. Human α-defensins HNP-1, -2 and -3, the best characterised members of human α-defensins, cause membrane permeabilisation via ion-permeable channel formation, induce DNA damage and suppress DNA synthesis in tumour cells [55–60]. HNPs are also cytolytic to normal human epithelial cells, fibroblasts and leukocytes [55, 60]. Interestingly, HBD-3 is also cytolytic to normal cells but at higher concentrations than to tumour cells. These data identify HBD-3 as the first human defensin demonstrated to display relatively selective, broad-ranged and direct anticancer activity.

The molecular basis of the specificity towards tumour cells by CAPs and defensins such as HBD-3, NaD1 and TPP3 [11, 12] is unclear but may be associated with morphological changes of plasma membranes upon tumour transformation to influence their robust growth, motility, invasion and metastasis, as opposed to normal cells [10]. Common features of tumour cells are their increased negatively-charged phospholipid [61, 62] and glycoprotein [63, 64] content in the membrane outer leaflet, increased membrane surface area [65] and membrane fluidity [66], all of which may contribute to enhanced affinity of membrane interaction, and CAP-mediated cytotoxicity. It is also interesting to note that PIPs, particularly PI(4,5)P_2_ and PI(3,4,5)_3_, have key signaling functions and crucial roles in the regulation of cell survival, growth, proliferation, invasion and metastasis, processes that are critical in tumourigenesis [67–71].

The finding that HBD-3 targets membrane lipids and permeabilises cells, as we have described previously for selected plant defensins [11, 12], suggests an evolutionarily conserved mechanism among membrane-targeting innate immune molecules that possess potential ‘phospholipid recognition patterns’. Indeed, the β2-β3 loop motif of HBD-3, NaD1 and TPP3 are reasonably conserved. Based on previous studies [11, 12], one might suggest that the positively-charged residues of HBD-3 would not only provide electrostatic attraction but also essentially involve an extensive H-bonding network with the anionic heads of phospholipids to stabilise defensin:lipid interactions. The last basic residue of the β2-β3 loop (i.e. K39 in HBD-3) is particularly important, as its mutation (to alanine) leads to abolished PI(4,5)P_2_ interaction, and ultimately membrane permeabilisation and anticancer activity. We also suggest that the hydrogen bond donor residues preceding the β2-β3 loop (i.e. K32 in HBD-3, H33 in NaD1) should be included as a possible extension of the ‘phospholipid recognition motif’. This is based on the structural conformation observation that H33 in NaD1 participates in hydrogen bonding at the defensin:lipid interface [11] and mutation thereof results in reduced PI(4,5)P_2_ binding as well as impaired defensin activity, together with the finding that HBD-3(K32A) has reduced lipid binding and cell permeabilisation activity.

It is also possible that HBD-3 dimerises and adopts a similar cationic grip to the plant defensins to accommodate PI(4,5)P_2_. In fact, Schibli *et al*. [22] proposed a dimer model of HBD-3 based on NMR data, although this was not consistent with the NaD1 or TPP3 ‘cationic grip’ PIP-binding dimeric conformation. Nevertheless, HBD-3 might undergo a conformational change upon lipid binding. It remains puzzling that, although NaD1 and TPP3 bind PI(4,5)P_2_ and oligomerise to form fibrils *in vitro*, a similar effect was not observed for HBD-3 binding of PI(4,5)P_2_ by transmission electron microscopy (data not shown). Interestingly, it was recently suggested using a molecular dynamics simulation that HBD-3 may be able to self-oligomerise (but was unable to lyse) in the context of a bacterial membrane surface [72]. Further study is therefore needed to determine whether oligomerisation is important for permeabilisation function of HBD-3.

In addition to PIPs, HBD-3 was also suggested to bind to other phospholipids such as PA and PS (Figure 5C). It was interesting to note that exogenously added PA actually enhanced the tumour cell permeability activity of HBD-3. It is possible that the exogenously added PA might have induced conformational change, promoted local accumulation or internalisation of HBD-3, thus enhancing its cytotoxicity. Previous studies have suggested that HBD-3 induces membrane damage in PS-enriched monocytes but not PS-deficient lymphocytes [33], implying that HBD-3 may interact with PS to mediate such effects. Indeed, in our study, HDB-3 was suggested to bind PS in protein-lipid overlay assays and exogenously added PS was shown to have a minor but significant inhibitory activity on the cell permeability function of HBD-3 (Figure 5C). However, in the context of antimicrobial function, it is tempting to speculate the ability of HBD-3 to interact with a diverse array of lipids, including PIPs, PA and PS, may impart the versatility to combat a wide range of different microbial pathogens.

Apart from the β2-β3 loop on HBD-3, NaD1 and TPP3, several other cationic motifs have been implied in phospholipid binding and functional importance. For example, the closely resembling β2-β3 loop (RGFRRR) loop on the plant defensin MtDef4 from *Medicago truncatula* is essential for fungal cell entry, mediated via PA binding [73]. The PI(3)P-binding RxLR motif carried by fungal pathogen effector molecules such as Avr is also important for plant cell internalisation [74, 75]. The cysteine-flanked KNKEKK segment of serum protein β_2_-glycoprotein 1 crucially binds to cardiolipin, enabling its role in anti-cardiolipin-mediated thrombosis, which is completely abolished by triple mutation KNGEGG [76, 77]. Likewise, deletion of the highly-conserved polybasic consensus Kx(R/K)xxKQKxK(R/K/Q)(R/K) from the membrane-targeting human Cd42 GTPase-activating protein results in impaired PI(3,4,5)P_3_ interaction and consequently, abolishes Cd42 activity *in vivo* [78]. Furthermore, although most PIP recognition and binding domains share little sequence similarities, the small cysteine-rich Zn^2+^-binding ‘Fab1, YOTB, Vac1, EEA1’ (FYVE) domains exhibit the most obvious (R/K)(R/K)HHCR pattern within their binding pocket for PI(3)P [79]. Together with our findings, it is suggested that an understanding of these patterns/codes may enable prediction of phospholipid binding partners and functional importance, and the design of phospholipid-targeted therapeutics.

In conclusion, we have demonstrated PI(4,5)P_2_-mediated membrane permeabilisation of tumour cells by HBD-3 via a conserved cationic loop motif. The interaction between HBD-3 and PI(4,5)P_2_ suggests a mechanistic conservation among defensins of different species, Indeed, the targeting of membrane lipids by defensins may well be a universal function for this family of innate defense peptides in their activity against various pathogens and could also explain the long-standing question as to why many CAPs have anti-tumour cell activity. It remains of significant interest to determine whether other defensins, particularly the many human defensins with unknown function, also use this mechanism of action in host defense against pathogens and altered-self such as tumour cells.

## MATERIALS AND METHODS

### Cell lines and cultures

Human epithelial cervical cancer (HeLa), leukemic monocyte lymphoma (U937), prostate cancer (PC3), promyelocytic leukemia (HL-60), leukemic T cell lymphoblast (Jurkat) cells were cultured in RPMI-1640 medium supplemented with 5–10% (v/v) fetal calf serum (FCS), 100 U/mL penicillin and 100 μg/mL streptomycin (Invitrogen, Carlsbad, CA). Human umbilical vein epithelial cell (HUVEC) cells were cultured in M199 medium (Invitrogen) supplemented with 20% (v/v) FCS, 40 μg/mL gentamicin, 4 μg/mL endothelial cell growth factor, 4 μg/mL L-glutamine and 135 μg/mL heparin, in 0.1% (w/v) gelatin-coated Corning CELLBIND flask (Corning Life Sciences, Acton, MA). Adult human dermal fibroblast (AHDF) and human coronary artery smooth muscle cell (CASMC) cells were cultured using FGM™-2 BulletKit™ and SmGM™-2 BulletKit™ (Lonza, Walkersville, MD). All cell lines were cultured at 37°C in a humidified atmosphere containing 5% CO_2_.

### Expression of HBD-3 and mutants in Pichia pastoris

HBD-3, HBD-3(K32A) and HBD-3(K39A) were cloned and recombinantly expressed in the methylotrophic yeast *P. pastoris*, and subsequently purified using SP-Sepharose as described previously [80].

### Cell viability assay

Different concentrations of HBD-3 were added to cells seeded in 96-well plates in appropriate complete medium. Initial plating cell density was pre-optimised to avoid confluence at endpoint. After 48 h, cell viability was determined using 3-(4,5-dimethylthiazol-2-yl)-2,5-diphenyltetrazolium bromide (MTT) (Sigma-Aldrich, MO) for adherent cells (HeLa, PC3, HUVEC, AHDF, CASMC) or 3-(4,5-dimethylthiazol-2-yl)-5-(3-carboxymethoxyphenyl)-2-(4-sulfophenyl)-2H-tetrazolium (MTS) (Promega, Madison, WI) coupled with phenzine methosulfate (PMS) (Promega) for suspension cells (U937, HL-60, Jurkat) prior to absorbance measurement at 570 nm and 490 mm, respectively. Absorbance readings of untreated control wells was designated as 100% cell viability.

### Propidium iodide uptake assay

Cells suspended at 1×10^6^ cells/mL in serum-free medium containing 0.1% (w/v) bovine serum albumin (BSA) (Sigma-Aldrich) were treated with HBD-3 at 37°C for 30 min. Nucleic acid stain propidium iodide (PI) was then added to a final concentration of 1 μg/mL and cells subjected to flow cytometry analysis using BD FACSCanto II Flow Cytometer and BD FACSDiva Software v6.1.1 (BD Biosciences, San Jose, CA). For each flow cytometry reading, 10,000 cells, gated appropriately based on forward scattering and side scattering, were recorded. The resultant data were processed using FlowJo software (Tree Star, San Carlos, CA) to determine PI-positivity, which reflects the level of membrane permeabilisation. For lipid inhibition assays, 15 μM HBD-3 was pre-incubated with 50 μM synthetic lipids, including L-α-phosphatidic acid (PA), L-α-phosphatidylcholine (PC), L-α-phosphatidylethanolamine (PE), L-α-phosphatidylserine (PS), L-α-phosphatidylinositol-4-phosphate (PI(4)P), L-α-phosphatidylinositol-3,5-bisphosphate (PI(3,5)P_2_), L-α-phosphatidylinositol-4,5-bisphosphate (PI(4,5)P_2_) and L-α-phosphatidylinositol-3,4,5-trisphosphate (PI(3,4,5)P)_3_. All lipids were sourced from Avanti Polar Lipids, Alabaster, AL. For neomycin inhibition experiments, U937 cells at 1×10^6^ cells/mL were pre-treated with 10 mM neomycin (Sigma-Aldrich), followed by three centrifugal washes with phosphate buffered saline (PBS) at 500 *g* for 5 min.

### ATP release assay

ATP release assay was conducted using an ATP bioluminescence assay kit (Roche Diagnostics, Mannheim, Germany). U937 and HeLa cells were suspended at 1×10^6^ cells/mL in PBS containing 0.1% (w/v) BSA, and mixed with luciferase/luciferin reagent at a ratio of 4:5. The mixture was added to HBD-3 samples and the level of ATP release measured immediately as bioluminescence emission signal intensity for 30 min with 30 s intervals.

### Labeling of HBD-3 with BODIPY FL EDA

Lyophilised HBD-3 was resuspended to 5 mg/mL in activation buffer (0.1 M MES, 0.5 M NaCl, pH 6). Ten-fold and 25-fold molar excess of 1-ethyl-3-(3-dimethylaminopropyl) carbodiimide and N-hydroxysulfosuccinimide (Thermo Scientific Pierce, Rockford, IL) was then added, respectively. After 15 min incubation, the pH was adjusted to 7.2 using 20× PBS, prior to reaction with five-fold molar excess of BODIPY FL EDA (Molecular Probes, Eugene, OR) for 3 h. Labeled HBD-3 was then purified from free label using a PD-10 desalting column (GE Healthcare, UK).

### Confocal laser scanning microscopy

Live imaging was performed on a Zeiss LSM-780 confocal microscope using a 63× oil immersion objective in a 37°C incubator with 5% CO_2_. Adherent HeLa cells were cultured overnight on coverslips while suspension U937 cells were immobilised onto 0.01% (w/v) poly-L-lysine-coated coverslips. Both cell types were prepared in serum-free RPMI 1640 medium containing 0.1% (w/v) BSA and 2 μg/mL PI. HBD-3 or BODIPY FL EDA-labeled HBD-3 was added directly to the imaging chamber to final concentration of 15 μM, via a capillary tube. In certain experiments, prior to imaging, cells were pre-stained with the membrane dye PKH67 as per manufacturer's instructions (Sigma-Aldrich) or transfected with expression vectors carrying GFP only or GFP-tagged Pleckstrin homology domain of phospholipase C-delta 1 (GFP-PH(*PLCδ*1)) (kindly provided by Christina Mitchell, Monash University, Australia), using Lipofectamine 3000 reagent (Invitrogen) as per manufacturer's instructions. For CLSM involving transfections, images were taken as 4×4 tiles over 30 min, and the resultant data for GFP and GFP-PH transfected cells were analysed by counting PI-positive cells.

### Protein-lipid overlay assay

Protein-lipid overlay assays using Membrane strip™ or PIP strip™ (Echelon Biosciences, Salt Lake City, UT) were performed using 1 μg/mL proteins as described previously [11]. Lipid binding was immunodetected using a combination of rabbit anti-HBD-3 IgG (50 μg/mL) and horseradish peroxidase-conjugated donkey-anti-rabbit Ig (10 μg/mL) antibodies. Chemiluminescence signal intensity was quantitated by densitometry analysis using ImageJ (National Institutes of Health, Bethesda, MD; http://imagej.nih.gov/ij/).

### Anti-PI(4,5)P_2_ antibody blocking assay

U937 cells at 1×10^6^ cells/mL were treated with 10 mM neomycin for 3 h and/or 15 μM defensins for 30 min in serum-free RPMI 1640 medium containing 0.1% BSA, followed by 20 min fixation with 2% (v/w) paraformaldehyde (Sigma-Aldrich), 10 min lysis with 0.5% (w/v) saponin (Sigma-Aldrich), and then 30 min blocking with 3% (w/v) BSA. Three 5-min centrifugal washes with PBS at 500 *g* were also included between steps. FITC-conjugated mouse anti-PI(4,5)P_2_ IgM (Echelon Biosciences) or IgM control (GeneTex, Irvine, CA) was subsequently used at 10 μg/mL for 30 min prior to flow cytometry analysis.

### ATP-encapsulated liposome leakage assay

Liposomes were generated as described previously [81] using natural PI(4,5)P_2_ (porcine brain, in chloroform:methanol:water at 20:9:1 molar ratio) and PC (chicken egg, in chloroform) purchased from Avanti Polar Lipids. PC only or PC:PI(4,5)P_2_ (95:5 molar ratio) solutions were dried under a stream of nitrogen gas followed by overnight vacuum-drying. The lipid films were rehydrated to a final concentration of 5 mg/mL in 20 mM HEPES (pH 7.2) containing 5 mg/mL adenosine-5-triphosphate disodium salt (ATP; Sigma-Aldrich) at 37°C for 1 h. After three subsequent cycles of freezing (liquid nitrogen) and thawing (25°C), multilamellar liposomes were extruded 15–20 times through a mini-extruder (Avanti Polar Lipids). Free ATP was removed by three centrifugal washes with 20 mM HEPES at 16,500 *g* prior to the ATP release assay. Liposomes treated with 1% Triton X-100 were included as positive control and assigned as total lysis, whereas HEPES only control served as background reading. The level of ATP release at a particular time point was determined by quotient of the corrected reading (after subtracting background) of the sample at that time point and corrected reading of total lysis, as per the following equation:

$$\%\;{\text{Lysis}}_{t}=\frac{{\text{Sample}}_{t}-{\text{HEPES}}_{t}}{\text{Triton }X-\text{HEPES}}\times 100$$

## References

[1] Boman HG (1995). Peptide antibiotics and their role in innate immunity. Annu Rev Immunol.

[2] Broekaert WF, Terras FR, Cammue BP, Osborn RW (1995). Plant defensins: novel antimicrobial peptides as components of the host defense system. Plant Physiol.

[3] Ganz T (2003). Defensins: antimicrobial peptides of innate immunity. Nat Rev Immunol.

[4] Brogden KA (2005). Antimicrobial peptides: pore formers or metabolic inhibitors in bacteria?. Nat Rev Microbiol.

[5] Jenssen H, Hamill P, Hancock RE (2006). Peptide antimicrobial agents. Clin Microbiol Rev.

[6] Gwyer Findlay E, Currie SM, Davidson DJ (2013). Cationic host defence peptides: potential as antiviral therapeutics. BioDrugs.

[7] Ulm H, Wilmes M, Shai Y, Sahl HG (2012). Antimicrobial host defensins-specific antibiotic activities and innate defense modulation. Front Immunol.

[8] Gaspar D, Veiga AS, Castanho MA (2013). From antimicrobial to anticancer peptides. A review. Front Microbiol.

[9] Mulder KC, Lima LA, Miranda VJ, Dias SC, Franco OL (2013). Current scenario of peptide-based drugs: the key roles of cationic antitumor and antiviral peptides. Front Microbiol.

[10] Hoskin DW, Ramamoorthy A (2008). Studies on anticancer activities of antimicrobial peptides. Biochim Biophys Acta.

[11] Poon IKH, Baxter AA, Lay FT, Mills GD, Adda CG, Payne JA, Phan TK, Ryan GF, White JA, Veneer PK, van der Weerden NL, Anderson MA, Kvansakul M (2014). Phosphoinositide-mediated oligomerization of a defensin induces cell lysis. eLife.

[12] Baxter AA, Richter V, Lay FT, Poon IK, Adda CG, Veneer PK, Phan TK, Bleackley MR, Anderson MA, Kvansakul M, Hulett MD (2015). The tomato defensin TPP3 binds phosphatidylinositol (4,5)-bisphosphate via a conserved dimeric cationic grip conformation to mediate cell lysis. Mol Cell Biol.

[13] Di Paolo G, De Camilli P (2006). Phosphoinositides in cell regulation and membrane dynamics. Nature.

[14] Balla T (2013). Phosphoinositides: tiny lipids with giant impact on cell regulation. Physiol Rev.

[15] Falkenburger BH, Jensen JB, Dickson EJ, Suh BC, Hille B (2010). Phosphoinositides: lipid regulators of membrane proteins. J Physiol.

[16] Harder J, Bartels J, Christophers E, Schroder JM (2001). Isolation and characterization of human beta-defensin-3, a novel human inducible peptide antibiotic. J Biol Chem.

[17] Garcia JR, Jaumann F, Schulz S, Krause A, Rodriguez-Jimenez J, Forssmann U, Adermann K, Kluver E, Vogelmeier C, Becker D, Hedrich R, Forssmann WG, Bals R (2001). Identification of a novel, multifunctional beta-defensin (human beta-defensin 3) with specific antimicrobial activity. Its interaction with plasma membranes of Xenopus oocytes and the induction of macrophage chemoattraction. Cell Tissue Res.

[18] Sorensen OE, Thapa DR, Roupe KM, Valore EV, Sjobring U, Roberts AA, Schmidtchen A, Ganz T (2006). Injury-induced innate immune response in human skin mediated by transactivation of the epidermal growth factor receptor. J Clin Invest.

[19] Harder J, Meyer-Hoffert U, Wehkamp K, Schwichtenberg L, Schroder JM (2004). Differential gene induction of human beta-defensins (hBD-1, -2, -3, and -4) in keratinocytes is inhibited by retinoic acid. J Invest Dermatol.

[20] Chen X, Niyonsaba F, Ushio H, Hara M, Yokoi H, Matsumoto K, Saito H, Nagaoka I, Ikeda S, Okumura K, Ogawa H (2007). Antimicrobial peptides human beta-defensin (hBD)-3 and hBD-4 activate mast cells and increase skin vascular permeability. Eur J Immunol.

[21] Schroeder BO, Wu Z, Nuding S, Groscurth S, Marcinowski M, Beisner J, Buchner J, Schaller M, Stange EF, Wehkamp J (2011). Reduction of disulphide bonds unmasks potent antimicrobial activity of human beta-defensin 1. Nature.

[22] Schibli DJ, Hunter HN, Aseyev V, Starner TD, Wiencek JM, McCray PB, Tack BF, Vogel HJ (2002). The solution structures of the human beta-defensins lead to a better understanding of the potent bactericidal activity of HBD3 against Staphylococcus aureus. J Biol Chem.

[23] Quinones-Mateu ME, Lederman MM, Feng Z, Chakraborty B, Weber J, Rangel HR, Marotta ML, Mirza M, Jiang B, Kiser P, Medvik K, Sieg SF, Weinberg A (2003). Human epithelial beta-defensins 2 and 3 inhibit HIV-1 replication. AIDS.

[24] Sun L, Finnegan CM, Kish-Catalone T, Blumenthal R, Garzino-Demo P, La Terra Maggiore GM, Berrone S, Kleinman C, Wu Z, Abdelwahab S, Lu W, Garzino-Demo A (2005). Human beta-defensins suppress human immunodeficiency virus infection: potential role in mucosal protection. J Virol.

[25] Leikina E, Delanoe-Ayari H, Melikov K, Cho MS, Chen A, Waring AJ, Wang W, Xie Y, Loo JA, Lehrer RI, Chernomordik LV (2005). Carbohydrate-binding molecules inhibit viral fusion and entry by crosslinking membrane glycoproteins. Nat Immunol.

[26] Pazgier M, Hoover DM, Yang D, Lu W, Lubkowski J (2006). Human beta-defensins. Cell Mol Life Sci.

[27] Feng Z, Jiang B, Chandra J, Ghannoum M, Nelson S, Weinberg A (2005). Human beta-defensins: differential activity against candidal species and regulation by Candida albicans. J Dent Res.

[28] Ferris LK, Mburu YK, Mathers AR, Fluharty ER, Larregina AT, Ferris RL, Falo LD (2013). Human beta-defensin 3 induces maturation of human langerhans cell-like dendritic cells: an antimicrobial peptide that functions as an endogenous adjuvant. J Invest Dermatol.

[29] Petrov V, Funderburg N, Weinberg A, Sieg S (2013). Human beta defensin-3 induces chemokines from monocytes and macrophages: diminished activity in cells from HIV-infected persons. Immunology.

[30] Funderburg N, Lederman MM, Feng Z, Drage MG, Jadlowsky J, Harding CV, Weinberg A, Sieg SF (2007). Human-defensin-3 activates professional antigen-presenting cells via Toll-like receptors 1 and 2. Proc Natl Acad Sci USA.

[31] Nagaoka I, Niyonsaba F, Tsutsumi-Ishii Y, Tamura H, Hirata M (2008). Evaluation of the effect of human beta-defensins on neutrophil apoptosis. Int Immunol.

[32] Sass V, Schneider T, Wilmes M, Korner C, Tossi A, Novikova N, Shamova O, Sahl HG (2010). Human beta-defensin 3 inhibits cell wall biosynthesis in Staphylococci. Infect Immun.

[33] Lioi AB, Rodriguez AL, Funderburg NT, Feng Z, Weinberg A, Sieg SF (2012). Membrane damage and repair in primary monocytes exposed to human beta-defensin-3. J Leukoc Biol.

[34] Rohrl J, Yang D, Oppenheim JJ, Hehlgans T (2010). Human beta-defensin 2 and 3 and their mouse orthologs induce chemotaxis through interaction with CCR2. J Immunol.

[35] Feng Z, Dubyak GR, Jia X, Lubkowski JT, Weinberg A (2013). Human beta-defensin-3 structure motifs that are important in CXCR4 antagonism. FEBS J.

[36] Lee AY, Phan TK, Hulett MD, Korner H (2015). The relationship between CCR6 and its binding partners: does the CCR6-CCL20 axis have to be extended?. Cytokine.

[37] Wang K, Wang JH, Baskaran H, Wang R, Jurevic R (2012). Effect of human beta-defensin-3 on head and neck cancer cell migration using micro-fabricated cell islands. Head Neck Oncol.

[38] Uraki S, Sugimoto K, Shiraki K, Tameda M, Inagaki Y, Ogura S, Kasai C, Nojiri K, Yoneda M, Yamamoto N, Takei Y, Nobori T, Ito M (2014). Human beta-defensin-3 inhibits migration of colon cancer cells via downregulation of metastasis-associated 1 family, member 2 expression. Int J Oncol.

[39] Schacht J (1976). Inhibition by neomycin of polyphosphoinositide turnover in subcellular fractions of guinea-pig cerebral cortex in vitro. J Neurochem.

[40] Schacht J (1978). Purification of polyphosphoinositides by chromatography on immobilized neomycin. J Lipid Res.

[41] Gabev E, Kasianowicz J, Abbott T, McLaughlin S (1989). Binding of neomycin to phosphatidylinositol 4,5-bisphosphate (PIP2). Biochim Biophys Acta.

[42] Arbuzova A, Martushova K, Hangyas-Mihalyne G, Morris AJ, Ozaki S, Prestwich GD, McLaughlin S (2000). Fluorescently labeled neomycin as a probe of phosphatidylinositol-4, 5-bisphosphate in membranes. Biochim Biophys Acta.

[43] Oren Z, Lerman JC, Gudmundsson GH, Agerberth B, Shai Y (1999). Structure and organization of the human antimicrobial peptide LL-37 in phospholipid membranes: relevance to the molecular basis for its non-cell-selective activity. Biochem J.

[44] Porcelli F, Verardi R, Shi L, Henzler-Wildman KA, Ramamoorthy A, Veglia G (2008). NMR structure of the cathelicidin-derived human antimicrobial peptide LL-37 in dodecylphosphocholine micelles. Biochemistry.

[45] Sood R, Domanov Y, Pietiainen M, Kontinen VP, Kinnunen PK (2008). Binding of LL-37 to model biomembranes: insight into target vs host cell recognition. Biochim Biophys Acta.

[46] Lee CC, Sun Y, Qian S, Huang HW (2011). Transmembrane pores formed by human antimicrobial peptide LL-37. Biophys J.

[47] Gauldie J, Hanson JM, Shipolini RA, Vernon CA (1978). The structures of some peptides from bee venom. Eur J Biochem.

[48] Killion JJ, Dunn JD (1986). Differential cytolysis of murine spleen, bone-marrow and leukemia cells by melittin reveals differences in membrane topography. Biochem Biophys Res Commun.

[49] Sui SF, Wu H, Guo Y, Chen KS (1994). Conformational changes of melittin upon insertion into phospholipid monolayer and vesicle. J Biochem.

[50] Eliassen LT, Berge G, Sveinbjornsson B, Svendsen JS, Vorland LH, Rekdal O (2002). Evidence for a direct antitumor mechanism of action of bovine lactoferricin. Anticancer Res.

[51] Mader JS, Salsman J, Conrad DM, Hoskin DW (2005). Bovine lactoferricin selectively induces apoptosis in human leukemia and carcinoma cell lines. Mol Cancer Ther.

[52] Eliassen LT, Berge G, Leknessund A, Wikman M, Lindin I, Lokke C, Ponthan F, Johnsen JI, Sveinbjornsson B, Kogner P, Flaegstad T, Rekdal O (2006). The antimicrobial peptide, lactoferricin B, is cytotoxic to neuroblastoma cells in vitro and inhibits xenograft growth in vivo. Int J Cancer.

[53] Yoo YC, Watanabe R, Koike Y, Mitobe M, Shimazaki K, Watanabe S, Azuma I (1997). Apoptosis in human leukemic cells induced by lactoferricin, a bovine milk protein-derived peptide: involvement of reactive oxygen species. Biochem Biophys Res Commun.

[54] Yoo YC, Watanabe S, Watanabe R, Hata K, Shimazaki K, Azuma I (1997). Bovine lactoferrin and lactoferricin, a peptide derived from bovine lactoferrin, inhibit tumor metastasis in mice. Jpn J Cancer Res.

[55] Lichtenstein A, Ganz T, Selsted ME, Lehrer RI (1986). In vitro tumor cell cytolysis mediated by peptide defensins of human and rabbit granulocytes. Blood.

[56] Lichtenstein AK, Ganz T, Nguyen TM, Selsted ME, Lehrer RI (1988). Mechanism of target cytolysis by peptide defensins. Target cell metabolic activities, possibly involving endocytosis, are crucial for expression of cytotoxicity. J Immunol.

[57] Muller CA, Markovic-Lipkovski J, Klatt T, Gamper J, Schwarz G, Beck H, Deeg M, Kalbacher H, Widmann S, Wessels JT, Becker V, Muller GA, Flad T (2002). Human alpha-defensins HNPs-1, -2, and -3 in renal cell carcinoma: influences on tumor cell proliferation. Am J Pathol.

[58] Lichtenstein A (1991). Mechanism of mammalian cell lysis mediated by peptide defensins. Evidence for an initial alteration of the plasma membrane. J Clin Invest.

[59] Kagan BL, Selsted ME, Ganz T, Lehrer RI (1990). Antimicrobial defensin peptides form voltage-dependent ion-permeable channels in planar lipid bilayer membranes. Proc Natl Acad Sci USA.

[60] Nishimura M, Abiko Y, Kurashige Y, Takeshima M, Yamazaki M, Kusano K, Saitoh M, Nakashima K, Inoue T, Kaku T (2004). Effect of defensin peptides on eukaryotic cells: primary epithelial cells, fibroblasts and squamous cell carcinoma cell lines. J Dermatol Sci.

[61] Ran S, Thorpe PE (2002). Phosphatidylserine is a marker of tumor vasculature and a potential target for cancer imaging and therapy. Int J Radiat Oncol Biol Phys.

[62] Utsugi T, Schroit AJ, Connor J, Bucana CD, Fidler IJ (1991). Elevated expression of phosphatidylserine in the outer membrane leaflet of human tumor cells and recognition by activated human blood monocytes. Cancer Res.

[63] Blackhall FH, Merry CL, Davies EJ, Jayson GC (2001). Heparan sulfate proteoglycans and cancer. Br J Cancer.

[64] Hollingsworth MA, Swanson BJ (2004). Mucins in cancer: protection and control of the cell surface. Nat Rev Cancer.

[65] Yamazaki D, Kurisu S, Takenawa T (2005). Regulation of cancer cell motility through actin reorganization. Cancer Sci.

[66] Barnett RE, Furcht LT, Scott RE (1974). Differences in membrane fluidity and structure in contact-inhibited and transformed cells. Proc Natl Acad Sci U S A.

[67] Katso R, Okkenhaug K, Ahmadi K, White S, Timms J, Waterfield MD (2001). Cellular function of phosphoinositide 3-kinases: implications for development, homeostasis, and cancer. Annu Rev Cell Dev Biol.

[68] Engelman JA, Luo J, Cantley LC (2006). The evolution of phosphatidylinositol 3-kinases as regulators of growth and metabolism. Nat Rev Genet.

[69] Bunney TD, Katan M (2010). Phosphoinositide signalling in cancer: beyond PI3K and PTEN. Nat Rev Cancer.

[70] Yamaguchi H, Yoshida S, Muroi E, Kawamura M, Kouchi Z, Nakamura Y, Sakai R, Fukami K (2010). Phosphatidylinositol 4,5-bisphosphate and PIP5-kinase Ialpha are required for invadopodia ­formation in human breast cancer cells. Cancer Sci.

[71] Wang W, Eddy R, Condeelis J (2007). The cofilin pathway in breast cancer invasion and metastasis. Nat Rev Cancer.

[72] Zhao X, Yu H, Yang L, Li Q, Huang X (2015). Simulating the antimicrobial mechanism of human beta-defensin-3 with coarse-grained molecular dynamics. J Biomol Struct Dyn.

[73] Sagaram US, El-Mounadi K, Buchko GW, Berg HR, Kaur J, Pandurangi RS, Smith TJ, Shah DM (2013). Structural and functional studies of a phosphatidic acid-binding antifungal plant defensin MtDef4: identification of an RGFRRR motif governing fungal cell entry. PLoS One.

[74] Kale SD, Gu B, Capelluto DG, Dou D, Feldman E, Rumore A, Arredondo FD, Hanlon R, Fudal I, Rouxel T, Lawrence CB, Shan W, Tyler BM (2010). External lipid PI3P mediates entry of eukaryotic pathogen effectors into plant and animal host cells. Cell.

[75] Yaeno T, Li H, Chaparro-Garcia A, Schornack S, Koshiba S, Watanabe S, Kigawa T, Kamoun S, Shirasu K (2011). Phosphatidylinositol monophosphate-binding interface in the oomycete RXLR effector AVR3a is required for its stability in host cells to modulate plant immunity. Proc Natl Acad Sci USA.

[76] Borchman D, Harris EN, Pierangeli SS, Lamba OP (1995). Interactions and molecular structure of cardiolipin and beta 2-glycoprotein 1 (beta 2-GP1). Clin Exp Immunol.

[77] Sheng Y, Sali A, Herzog H, Lahnstein J, Krilis SA (1996). Site-directed mutagenesis of recombinant human beta 2-glycoprotein I identifies a cluster of lysine residues that are critical for phospholipid binding and anti-cardiolipin antibody activity. J Immunol.

[78] Karimzadeh F, Primeau M, Mountassif D, Rouiller I, Lamarche-Vane N (2012). A stretch of polybasic residues mediates Cdc42 GTPase-activating protein (CdGAP) binding to phosphatidylinositol 3,4,5-trisphosphate and regulates its GAP activity. J Biol Chem.

[79] Kutateladze T, Overduin M (2001). Structural mechanism of endosome docking by the FYVE domain. Science.

[80] Lay FT, Mills GD, Poon IK, Cowieson NP, Kirby N, Baxter AA, van der Weerden NL, Dogovski C, Perugini MA, Anderson MA, Kvansakul M, Hulett MD (2012). Dimerization of plant defensin NaD1 enhances its antifungal activity. J Biol Chem.

[81] Zhang L, Rozek A, Hancock RE (2001). Interaction of cationic antimicrobial peptides with model membranes. J Biol Chem.

